# Whole-Genome Sequence of *Escherichia coli* Serotype O157:H7 Strain EDL932 (ATCC 43894)

**DOI:** 10.1128/genomeA.00647-16

**Published:** 2016-07-14

**Authors:** Gaylen A. Uhlich, George C. Paoli, Chin-Yi Chen, Bryan J. Cottrell, Xinmin Zhang, Xianghe Yan

**Affiliations:** a Molecular Characterization of Foodborne Pathogens Research Unit, U.S. Department of Agriculture, Agriculture Research Service, Eastern Regional Research Center, Wyndmoor, Pennsylvania, USA; b BioInfoRx, Inc., Madison, Wisconsin, USA

## Abstract

The genome sequence of *Escherichia coli* serotype O157:H7 EDL933, a ground beef isolate from a 1983 hemorrhagic colitis outbreak, is a standard reference for comparative genomic studies of Shiga toxin-producing *E. coli* strains. Here, we report the genome sequence of a patient stool isolate from that outbreak, strain EDL932.

## GENOME ANNOUNCEMENT

Shiga toxin-producing *Escherichia coli* (STEC) strains cause intestinal disease characterized by hemorrhagic colitis, which may progress to severe sequelae, such as hemolytic-uremic syndrome. In the United States, O157:H7 is the most important STEC serotype, both in total numbers of individual cases and large outbreaks.

STEC O157:H7 strain EDL933 (CDC; aka ATCC 43895) is a ground beef isolate from a 1983 hemorrhagic colitis outbreak in Michigan (1). The closed EDL933 sequence (GenBank accession no. AE005174.2 [2]) and a newly annotated version (GenBank accession no. CP008957 [3]) have served as reference genomes for sequence comparisons with many STEC strains.

Individual serotype O157:H7 strains show significant genotypic variability depending on the environment from which they were isolated. This is evident with regard to biofilm-forming properties and RpoS-dependent stress resistance (4, 5). Here, we report the whole-genome sequencing (WGS) of strain EDL932 (ATCC 43894), a patient isolate from that same 1983 Michigan outbreak.

Large genomic DNA (gDNA) fragments for single-molecule real-time (SMRT) sequencing were extracted from a frozen cell pellet (5 ml of overnight culture) of EDL932, designated 43894OW, using the Qiagen Genomic-tip 100/G kit. SMRT sequencing was done at the University of Delaware Sequencing and Genotyping Center (Newark, DE) using the PacBio RSII SMRT DNA sequencing system. *De novo* assembly of the SMRT reads used Hierarchical Genome Assembly Process (version 3). Total DNA was also sent to ProteinCT Biotechnologies, LLC (Madison, WI) for complete WGS workflow. Libraries were prepared using the Nextera DNA library preparation kit (Illumina) and sequenced using Illumina MiSeq. Approximately 4.5 million 2 × 250-bp paired-end reads were generated. Raw data quality was evaluated using FastQC, and Trimmomatic (USADELLAB.org) was used to remove adapters and low-quality sequences (<Q20). Clean data were assembled with Velvet (https://www.ebi.ac.uk/~zerbino/velvet/ [6]). Several parameters were tested, and the assemblies were evaluated by comparison to the EDL933 NCBI genome reference sequence (accession no. NZ_CP008957.1). The final assembly used in the report has a hash size of 69. In addition to genome assembly, clean reads were remapped to the EDL933 NCBI genome using Burrows-Wheeler Aligner (BWA) (7). Duplicated reads were removed with Picard (http://broadinstitute.github.io/picard/); variants were called using SAMtools (8) and annotated with SnpEff (9).

The unclosed 43894OW draft genome aligned with the 5,547,323-bp EDL933 reference genome, except for a nearly 13-kbp region with exceptional coverage depth, indicating gene duplication in strain EDL932 (Integrative Genomics Viewer [IGV]; Broad Institute, Cambridge, MA). Complete resolution of duplicated areas may expand the genome size. Variant detection identified small numbers of single-nucleotide polymorphisms (SNPs) and indels, many with low-quality scores. Included was a T-to-G transversion at position 721 of the *rpoS* coding sequence (CDS) compared to the reference EDL933, which changes a stop codon to a glutamic acid residue and extends EDL932 RpoS to 330 residues. This transversion matched the consensus for other serotype O157 strains, including strain EDL933 (ATCC 43895) *rpoS* gene sequenced in our earlier study (10), indicating variability among different outbreak isolates or a mistake during the original EDL933 sequence.

### Nucleotide sequence accession numbers.

This whole-genome shotgun project has been deposited at DDBJ/ENA/GenBank under the accession no. LPWC00000000. The version described in this paper is version LPWC02000000.
